# Flexural and Viscoelastic Properties of FRP Composite Laminates under Higher Temperatures: Experiments and Model Assessment

**DOI:** 10.3390/polym14112296

**Published:** 2022-06-05

**Authors:** Getahun Tefera, Sarp Adali, Glen Bright

**Affiliations:** Discipline of Mechanical Engineering, University of KwaZulu-Natal, Durban 4041, South Africa; adali@ukzn.ac.za (S.A.); brightg@ukzn.ac.za (G.B.)

**Keywords:** hybrid and non-hybrid laminate, flexural performance, viscoelastic properties, failure modes, empirical models

## Abstract

This study investigates an experimental and analytical study on the flexural, failure, and viscoelastic properties of hybrid and non-hybrid composite laminates at increasing temperatures and frequencies. Carbon, glass, and hybrids of the two fibre materials with stacking sequences of [0/90]_s_ were considered, and specimens were prepared via the resin transfer moulding method. Three-point bending and dynamic mechanical analysis tools were used. The failure surfaces of the laminates were examined using a scanning electron microscope. The results indicated that the flexural strength, modulus, and strain at failure of all groups of laminates decreased as the temperature increased. In particular, the storage modulus, damping factor, flexural strength, and flexural modulus properties of all groups of laminates increased as the hybrid ratio decreased on each targeted temperature and frequency test. However, the strain at failure increased as the hybrid ratio increased. Additionally, results obtained from the scanning electron microscope images confirmed that combinations of delamination and debonding failure modes were observed on the stacking sequences of [0]_s_ and [90]_s_ layers of bidirectional laminates. Finally, a comparison between the storage modulus results of all groups of laminates was conducted with three empirical models. The empirical model developed by Gibson et al. provided the most accurate prediction for all groups of laminates in the targeted temperature and frequency range. The predictions using the remaining empirical models were broadly similar. Further work is needed to optimise the empirical parameters and minimise the errors.

## 1. Introduction

Fibre-reinforced polymer (FRP) composite materials are widely used in the manufacturing of wind turbine blades, aerospace, automotive, and marine structural components, and in the construction industry [1,2], due to their advantages in high strength, stiffness, lightness, ease to form intricate shapes, and good corrosion resistance behaviour. Mostly, glass-fibre-reinforced polymer (GFRP) is the preferred FRP material, due to economic reasons [3]. Applications of carbon-fibre-reinforced polymer (CFRP) composite materials in horizontal-axis wind turbine blades have attracted the interest of many manufacturing industries due to ensuring the highest stiffness on large blades and reducing weight [4]. However, the failure strain is low, and it has higher costs compared to glass fibre [5,6]. Glass fibres have lower stiffness and a higher strain-to-failure behaviour compared to carbon fibres [7]. Particularly, carbon-fibre composite has a lower compressive-to-tensile strength ratio, which is one of the disadvantages of the material when used for composite structural components exposed to flexural and compressive loading [8]. Hybrid structures have been designed using low-elongation materials (e.g., carbon fibre) and other high-elongation materials (e.g., glass fibre) to enhance the strain-to-failure behaviour and reduce cost [9,10]. Since carbon fibres are lighter than glass fibres, hybridisation of the two fibres is used to retain the advantages that lead to a lower material cost, high strength and stiffness, and a moderate weight [11].

Research on the mechanical behaviour of hybrid and non-hybrid epoxy composite materials is a key factor in the design and manufacturing of composite structural components applicable under different environmental conditions [12]. For example, in FRP composite structures that were exposed to flexural loading, compression on the upper layer and tensile stress on the lower layer occurred [13]. It is important to know the compressive and tensile behaviour of FRP materials before using them for composite structures. Particularly, the flexural behaviours of glass- and carbon-reinforced hybrid epoxy composite laminates were investigated extensively under room temperature tests in [14,15,16,17]. The flexural and tensile moduli of hybrid composite laminates were decreased as the hybrid ratio increased [18,19,20].

The performance of FRP composite materials at elevated working temperatures has been a serious concern that needs investigation before their incorporation into engineering structures [21,22]. Shenghu et al. [23] investigated the tensile behaviour of hybrid and non-hybrid FRP specimens subjected to elevated temperatures. Similarly, Ke et al. [24] conducted testing to assess the tensile performance and compressive strength of pultruded CFRP plates at elevated temperatures [25]. Moreover, Rami et al. [26] experimentally assessed the tensile strength and tensile modulus of CFRP, GFRP, and their hybrid composition when exposed to different testing temperatures. According to the authors’ findings, the performance of the specimens was decreased due to bonding loss, which typically confirms the temperature-dependent properties of FRP composite materials [27]. The tensile strength and elastic modulus of GFRP and basalt-fibre-reinforced polymer (BFRP) plates were tested at different temperatures [28,29]. The authors found that the performance of the plates was reduced significantly when the testing temperatures were above the glass transition temperature (T_g_) of the polymer matrix. The strength, stiffness, and density of FRP composite materials are better than those of steel at room temperature. Meanwhile, because of the temperature dependence of FRP composite materials, their mechanical behaviour varies when the working temperature increases. R.J.A. Hamad et al. [30] compared the mechanical properties of FRP bars and steel bars under elevated testing temperatures for use in the construction sector. The results indicated that FRP bars suffered significant reductions in their mechanical behaviours upon exposure to elevated temperatures up to 450 °C, at which point GFRP and BFRP melted and lost their tensile strength.

J.R. Correia et al. [31] conducted an experimental and theoretical investigation to assess the mechanical response of a GFRP pultruded profile at elevated testing temperatures. They used dynamic mechanical analysis (DMA) and differential scanning calorimetry (DSC) tools to determine the glass transition temperature and decomposition process of the material. Additionally, an empirical model was developed in [32,33] for predicting the elastic modulus and tensile strength of FRP composites when exposed to elevated temperatures under shear and flexural loading. Milad et al. [34] considered the effect of fibre length, orientation, and laminate thickness to assess the flexural and impact performance of FRP composite at elevated temperatures.

With the background described by the above authors, various experimental works of fundamental research have reported on the mechanical properties of FRP composite materials as a function of temperature. The most common FRP materials are carbon and glass fibres. In particular, the compressive, tensile, flexural, and viscoelastic properties of CFRP, GFRP, and hybrids of the two materials have been assessed experimentally under different testing temperatures. Results from all of the authors confirm the temperature-dependent behaviour of FRP materials. Additionally, empirical models have been developed by different authors to reduce the material and testing costs under elevated temperatures. Still, further research is needed on FRP materials to be used for different applications.

Today, the structures of small, medium, and large horizontal-axis wind turbine (HAWT) blades are developed using FRP composite materials. In particular, large HAWT blade manufacturing industries use CFRP materials to optimise stiffness. Unidirectional and bidirectional fibres are preferred on the spar caps and skin sections to reduce the bending and torsion loads of the blades [35]. The components of HAWT blades are exposed to different environmental conditions during their lifetime. Temperature variations may affect the mechanical properties and lifetime of the FRP composite materials that were used to manufacture the different components of the blades.

In this study, the flexural and viscoelastic properties of bidirectional epoxy hybrid composite laminates reinforced with E-glass and T-300 carbon fibres—which were used on the skin section of the blades—were characterised by a three-point static bend test and a DMA tool at different testing temperatures. The fibres’ orientation and hybrid ratios were considered as a parameter to study the flexural and viscoelastic behaviour of the materials. The glass transition temperature and decomposition process were determined using the DMA tool. Failure behaviour under room temperature tests was observed using scanning electron microscopy (SEM). Additionally, the storage modulus results obtained from experimental tests were validated with the existing empirical models developed by different researchers as a function of temperature and frequency. Finally, the errors were compared, and the results obtained were within the acceptable ranges.

## 2. Experimental Program

This section presents the properties of constituent materials and details of the composite laminates’ preparation process and testing methods. A series of laminates were tested via three-point bending and DMA to investigate their viscoelastic behaviour and flexural performance under higher temperatures.

### 2.1. Material Properties

Laminates were prepared using T-300 carbon fibre, E-glass fibre, prime 27 LV epoxy resin, and prime 27 LV slow-hardened resin purchased from AMT composites in Durban, South Africa. The properties of the fibres and the epoxy matrix at room temperature are shown in Table 1.

### 2.2. Laminate Preparation Details

Hybrid and non-hybrid epoxy composite laminates were produced using a resin transfer moulding (RTM) process. The composite laminates were arranged bidirectionally with stacking sequences of [0/90]_s_ and divided into four groups of carbon (C), glass (G), and glass–carbon fibre hybrid (GC and GCG) laminates for testing under different temperatures. Initially, peel ply, carbon fibres, glass fibres, spiral binders, core mats, and infusion mesh were laid out on a glass table. Next, the mould was properly sealed using a vacuum bag and a vacuum was created. Then, the epoxy resin was impregnated into the mould using a vacuum pump. Finally, the prepared bidirectional laminates were cured on a glass table at ambient temperature (25 °C) for 24 h, and then demoulded and post-cured in an oven for 16 h at 65 °C.

**Figure 1:**
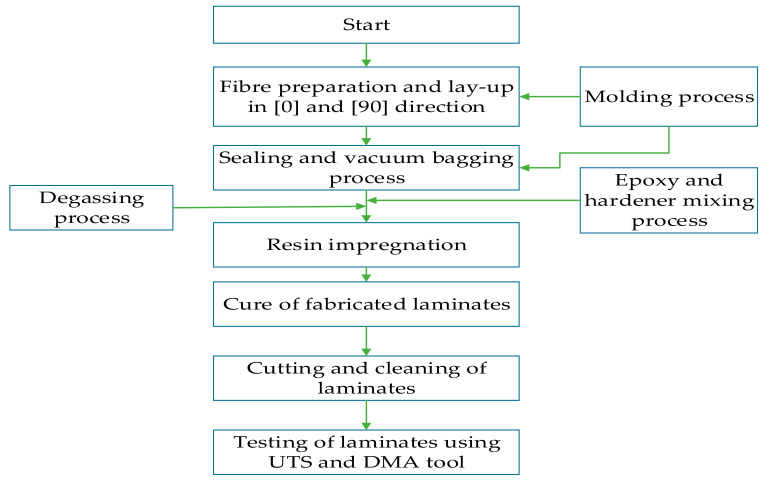
Process flowchart used in the manufacturing and testing of laminates using the RTM process.

Laminates were cut using a computer numerical control (CNC) machine within a tolerance of 0.02 mm to prepare them for testing. A total of 116 laminates were prepared for the three-point bending and DMA tests. Before the test, the laminates were cleaned and flashes were removed using sandpaper. The laminate preparation and testing process using RTM is shown in Figure 1.

**Figure 2:**
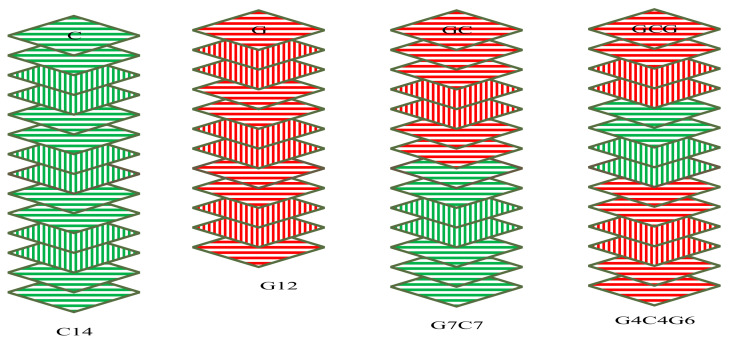
Orientations and stacking sequences of carbon, glass, and hybrids of the two fibres.

In this study, the fibre volume fractions of all laminates were determined to be 55% using the matrix digestion (burn-off) test according to the specifications of ASTM 3171-99. Fibre orientations and stacking sequences considered to produce bidirectional composite laminates are shown in Figure 2. For characterisation, the hybrid ratio was considered to be 1 when the laminates were produced from pure glass-fibre layers and 0 when the samples were produced from pure carbon-fibre layers. The details of hybrid and non-hybrid composite laminates and their designation are shown in Table 2.

### 2.3. Three-Point Static Bending Test

Flexural tests on hybrid and non-hybrid composite laminates were carried out according to ASTM: D7264/D7264M-07 [37] using a three-point bending test at a span-to-depth ratio of 32 utilising a Lloyd LR30k testing machine. A minimum of five laminates were tested for each stacking sequence and configuration, at a constant crosshead speed of 1 mm/min, at 25, 50, 75, and 100 °C. The span, thickness, and width of laminates were considered to be 146.24 ± 0.96 mm, 4.57 ± 0.03 mm, and 13 ± 0.02 mm, respectively. The laminates were exposed to the targeted testing temperatures in a standard furnace for 2 h before a three-point bending test occurred. A HEATCON thermocouple was mounted to measure the test temperatures. The failure modes of the laminates after a three-point bending test were inspected and analysed using a scanning electron microscope (SEM).

### 2.4. Dynamic Mechanical Analysis (DMA)

DMA tests were carried out on hybrid and non-hybrid composite laminates as per ASTM: D5023, using a DMA Q 800 TA instrument. The heating rate was increased at 2 °C/min, and frequencies were set at 1 Hz, 10 Hz, and 100 Hz for each laminate. The glass transition temperature (T_g_) of epoxy resin was measured using a DMA tool. Liquid nitrogen was used as a cooling agent. The dimensions of the test samples were set at a thickness of 4.57 ± 0.03 mm, width of 13 ± 0.02 mm, and length of 64 ± 0.02 mm. In the DMA experiments, sensors measured the testing temperature and loading.

### 2.5. Scanning Electron Microscopy (SEM) Analysis

The flexural failure behaviour along the cross-sections of bidirectional carbon, glass, and glass–carbon and glass–carbon–glass hybrid composite specimens were observed using a scanning electron microscope (SEM). Before SEM observations, all bidirectional composite samples were coated with gold for approximately 5 min.

## 3. Test Results and Discussion

In this section, experimental results found from the three-point bending and DMA tests on composite laminates are presented and analysed in detail with regard to their flexural performance and viscoelastic properties at increasing temperature and frequency.

### 3.1. Flexural Response and Characterisation

**Figure 3:**
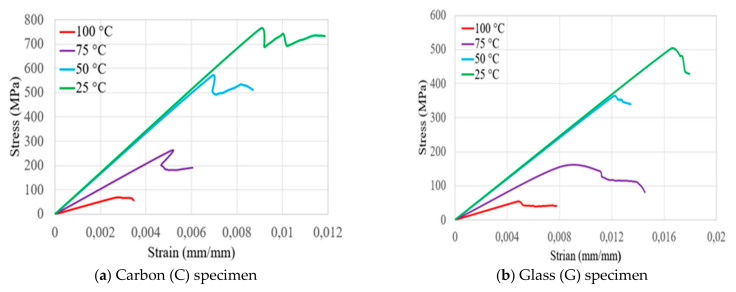

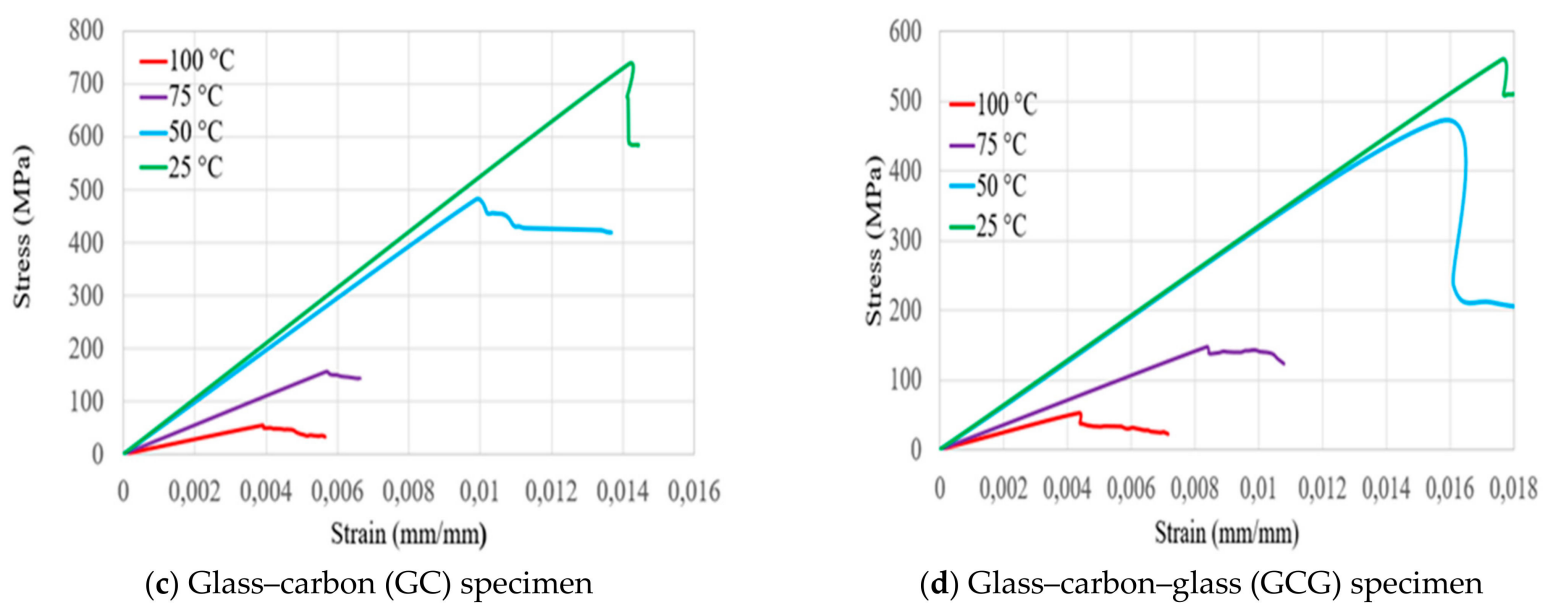
Stress–strain curves of the tested hybrid and non-hybrid composite specimens.

**Figure 4:**
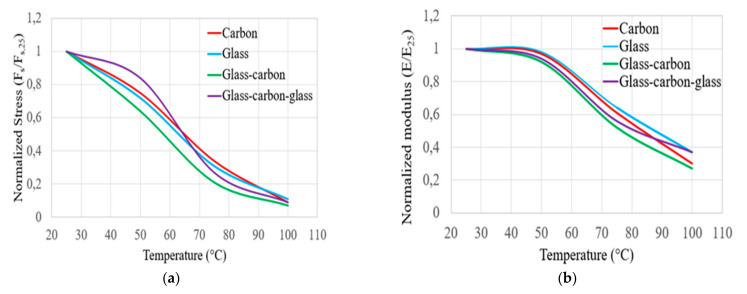
Comparison of the normalised flexural stress (**a**) and modulus (**b**) of hybrid and non-hybrid bidirectional composite specimens.

Figure 3 presents the flexural stress and strain relationships of bidirectional composite samples of carbon, glass, glass–carbon, and glass–carbon–glass laminates as a function of increasing temperature. It shows that the flexural strength of tested carbon (C), glass (G), glass–carbon (GC), and glass–carbon–glass (GCG) bidirectional composite laminates decreased as the testing temperatures increased. As Figure 4 indicates, linear deformations in all laminates were observed until they approached their maximum flexural stress. Once the laminates reached their maximum flexural strength, failure and a sudden drop in properties occurred. The average test results on the flexural strength (F_s_) and modulus (E) properties at different testing temperatures for the bidirectional C, G, GC, and GCG laminates are summarised in Table 3, Table 4, Table 5 and Table 6, respectively. Since the modulus of glass fibres is much lower than that of carbon fibres, both flexural strength and modulus decreased with increasing thickness of the glass fibre in the laminates. In the case of hybrid GC laminates, the thickness of the glass fibre was 51.54% of the total thickness of the laminates. When 51.54% glass fibre was applied on the outer upper surface of the laminates, the flexural strength and modulus decreased significantly when compared to carbon laminates. This occurred due to the presence of compressive and tensile stresses at the outer and bottom surfaces of the laminate.

The flexural properties of hybrid GCG laminates were assessed by placing 27.83% carbon fibre in the middle of the laminates. Table 6 and Figure 4d show that the flexural strength and modulus of the laminates were higher than those of the glass laminates according to the results obtained at 25 °C. In particular, the flexural strains of the hybrid laminates were optimised, making them nearly equal to those of the pure glass laminates during room temperature tests. Based on this, the thickness and position of carbon fibres in the laminates have a direct effect on the flexural strain, strength, and modulus values of the hybrid laminates.

In addition, those tables contain the ratios of flexural strength (F_s_/F_s,25_) and flexural modulus (E/E_25_) of all of the targeted tested laminates. Furthermore, the values of the coefficient of variation of all laminates were calculated from the tested specimens. The ranges of the coefficient of variation (CV) of the measured flexural strength of the tested C, G, GC, and GCG laminates were 3.09–7.37%, 2.58–7.87%, 1.8–5.33%, and 2.23–8.13%, respectively, at the targeted temperatures. However, the CV ranges of the measured flexural modulus of the tested C, G, GC, and GCG laminates were 0.85–7.35%, 3.77–7.62%, 3.91–7.80%, and 2.13–4.63%, respectively. The larger values of CV were observed in calculating the flexural strength and modulus of the hybrid GCG and GC laminates. In all groups of laminates, the value of CV was less than 10%, which is a statistically acceptable result.

The normalised flexural strength (F_s_/F_s,25_) and modulus (E/E_25_) properties of bidirectional carbon, glass, glass–carbon, and glass–carbon–glass laminates at increasing temperatures are shown in Figure 4a,b. Mainly, Figure 4a and Table 3, Table 4, Table 5 and Table 6 reveal that at 50 °C, the flexural strength of the C, G, GC, and GCG laminates was reduced by about 25%, 28%, 36%, and 18%, respectively, compared to the results obtained at room temperature (25 °C). The flexural strength of the C, G, GC, and GCG laminates decreased as the temperature approached 75 °C by approximately 66%, 69%, 79%, and 63%, respectively. In this case, the reduction in the flexural strength of the targeted laminates was higher as the epoxy matrix approached its glass transition temperature. The decrease in the flexural strength of the hybrid GC laminates was the most severe compared to the other laminates. This indicates that the GC laminates cannot maintain their flexural strength up to this temperature level. Similarly, the flexural modulus properties of the C, G, GC, and GCG composite laminates are illustrated in Figure 4b and Table 3, Table 4, Table 5 and Table 6, respectively. The test results show that the flexural modulus of the C, G, GC, and GCG composite laminates was reduced by about 3%, 2%, 8%, and 6%, respectively, as the testing temperature changed from 25 to 50 °C. Furthermore, the flexural modulus of the C, G, GC, and GCG composite laminates was reduced by about 70%, 63%, 73%, and 62%, respectively, at a temperature of 100 °C. Based on the normalised flexural strength and modulus results, it seems that the composite hybrid GCG laminate has better temperature resistance properties at 50 and 75 °C. However, the flexural strength and modulus properties of hybrid GC laminates are close to those of pure carbon laminates.

### 3.2. Dynamic Mechanical Analysis (DMA)-Based Characterisation

DMA tests were performed on C, G, GC, and GCG composite laminates to assess their mechanical responses as a function of temperature and frequency. The dynamic responses of those laminates were identified using the DMA tool, and their results are summarised in Table 7. It is clear from Table 7 that at a frequency of 1 Hz and 10 Hz, the values of the storage modulus, loss modulus, and damping ratio of all groups of laminates were generally similar. However, there was an increase in the values of the storage modulus, loss modulus, and damping factor as the testing frequency increased to 100 Hz. The similarity in the mechanical responses of all groups of laminates at 1 Hz and 10 Hz occurred due to the flow behaviour of the polymer matrix at low frequency, acting similarly to the flow of polymer at elevated temperatures. At 100 Hz, the gaps between the crosslinking of the polymer matrix tended to close on one another. This caused the composite laminates to behave elastically for a longer period.

**Figure 5:**
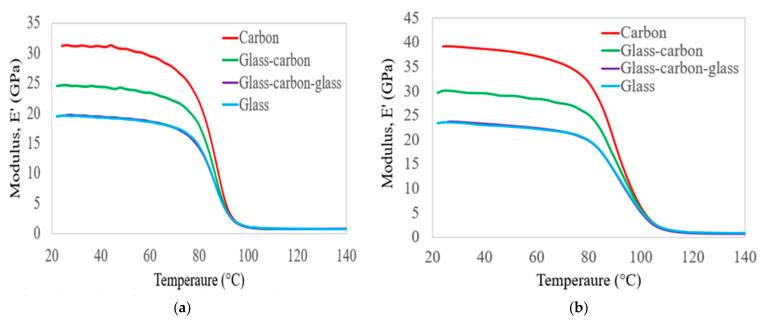
Comparison of the values of storage modulus on hybrid and non-hybrid bidirectional laminates at frequencies of (**a**) 1 Hz and (**b**) 100 Hz.

Figure 5 shows the comparison of the storage modulus results obtained at a frequency of 1 Hz and 100 Hz for the C, G, GC, and GCG laminates as a function of increasing temperature. It is clear from Figure 5 that the storage modulus results of all groups of laminates were increased from their initial values as the frequency changed from 1 Hz to 100 Hz. Those variations in storage modulus occurred below the glass transition temperature of the polymer matrix. However, the storage modulus of all groups of laminates was the same below the decomposition state within all of the targeted testing frequencies. This happened due to damage to the fibre–epoxy resin interface.

The storage modulus results of all groups of laminates at 50, 80, and 100 °C were compared to their room temperature values, considering frequencies of 1 Hz and 100 Hz. When the temperature was increased to 50 °C, the storage modulus of the C, G, GC, and GCG laminates changed from 2 to 4% at the targeted frequencies, compared to the room temperature values. Based on the storage modulus results, it seems that the composite hybrid laminates have nearly equal temperature resistance properties compared to pure carbon and glass laminates at a service load in strengthening the skin of the structure of the wind turbine blades. In the case of 80 °C and 1 Hz, the reduction in the storage modulus of the C, G, GC, and GCG laminates was 30%, 26%, 27%, and 28%, respectively, compared to the room temperature values. Those values were reduced to 19%, 16%, 16%, and 17%, respectively, as the frequency increased to 100 Hz. This indicates that the hybrid GC laminates can maintain their stiffness better than pure carbon fibre up to this temperature level. In particular, at 100 °C and 1 Hz, the storage modulus of the laminates changed from 95 to 97% compared to room temperature values, whereas the change reached from 77 to 84% for the cases of 100 Hz. Mainly, this change occurred due to the slow movement of polymers at the higher frequency of their elasticity properties for prolonged periods. The T_g_ values of the epoxy matrix at various frequencies are identified by the curves in Figure 5, which range from 80 to 86 °C.

**Figure 6:**
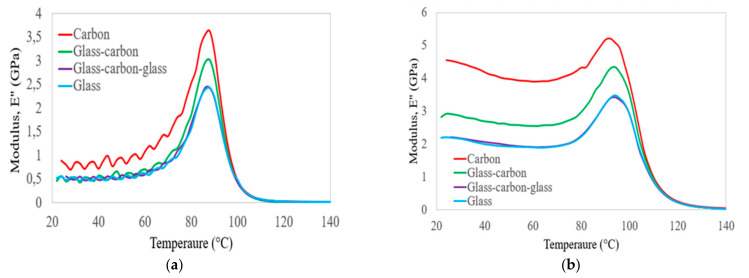
Comparison of the values of loss modulus on hybrid and non-hybrid bidirectional laminates at frequencies of (**a**) 1 Hz and (**b**) 100 Hz.

The comparison of the loss modulus results obtained on C, G, GC, and GCG laminates as a function of temperature and frequency is shown in Figure 6 and Table 7. In particular, it is clear from Figure 6 that the maximum loss modulus in all groups of laminates was observed at the T_g_ of the epoxy matrix. This occurred due to an increase in internal friction that enhanced the mobility of the epoxy matrix to dissipate heat [38]. The increase in the storage modulus values and T_g_ of all groups of laminates at increasing frequency occurred due to temperature-dependent molecular relaxation behaviour in polymers, i.e., molecular relaxation takes place at higher temperatures. The amount of dissipation energy on the four laminates can be assessed from the loss modulus results presented in Table 7. The lowest energy dissipation performance was identified in the hybrid GC laminates at 1 Hz. In the case of 100 Hz, pure glass and carbon laminates had the lowest and highest energy dissipation performance, respectively. However, hybridisation of the two materials was used to obtain the optimal energy dissipation performance at higher frequencies.

The skin and spar cap sections of the wind turbine blade structures were manufactured using bidirectional carbon, glass, and hybrids of the two materials to enhance their rigidity and reduce their weight. In particular, the blades’ exterior parts are required to withstand the torsion load; therefore, bidirectional fibres are needed to develop the structures [35]. Due to having the highest stiffness properties, carbon fibre is the most suitable material to develop the structures of large wind turbine blades. Hybrids of carbon and glass fibres can reduce the cost of carbon fibres. The exterior parts of the blade, such as the skin and spar cap sections, are affected by the fluctuating wind loads. It is necessary to know the damping properties of bidirectional hybrid and non-hybrid composite laminates with increasing temperature and frequency before using the materials to develop the blades’ exterior structures.

**Figure 7:**
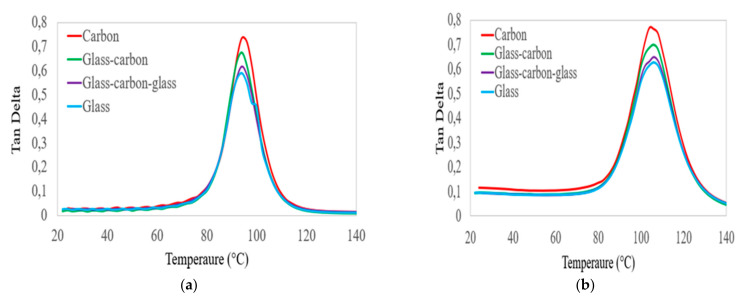
Comparison of the values of damping factor on hybrid and non-hybrid bidirectional laminates at frequencies of (**a**) 1 Hz and (**b**)100 Hz.

The damping ratios of the four laminates with increasing temperature and frequency were assessed in three separate temperature zones, as shown in the curves of Figure 7. The first range is considered below the T_g_ of the epoxy matrix. In this zone, the molecular chain mobility of the epoxy matrix did not change; thus, the damping ratios of all groups of laminates were largely similar, and slightly increased. The second zone includes the temperature approaching the T_g_ of the polymer matrix. In this zone, the molecular chain mobility increased. Thus, the values of the damping ratio in all groups of laminates were increased, and the hybrid GC laminates had a better damping ratio compared to glass laminates. The values of the damping ratio depend on the amount of carbon fibre in the hybrid laminates, and increased as the percentage of carbon fibre increased in the hybrid laminates. The third zone was considered to be below the decomposition temperature. In this zone, the resin softened and the bond between the fibre–matrix interface was damaged. The values of the damping ratio of all groups of laminates were largely similar and significantly reduced at the targeted frequency. Based on the DMA test results, all groups of laminates had a better storage modulus and lower energy dissipation performance below the glass transition temperature of the epoxy matrix. This property can be improved by incorporating higher-stiffness carbon fibre into lower-stiffness glass fibre in hybrid composite laminates.

### 3.3. Fracture Modes of Hybrid and Non-Hybrid Laminates

The common failure modes under flexural loading include compressive, tensile, and delamination failure modes. Compressive failure modes include microbuckling, kinking, and splitting. The failure of a hybrid laminate is dependent on the maximum bending moment experienced by the individual constituent material during a bending test. In particular, due to variations in failure strain across the thickness of a bidirectional hybrid composite, the critical location may not always lie on the surface ply [16]. After the flexural tests, failure modes of bidirectional hybrid and non-hybrid laminates on the fractured cross-sectional surfaces were observed using a scanning electron microscope (SEM).

**Figure 8:**
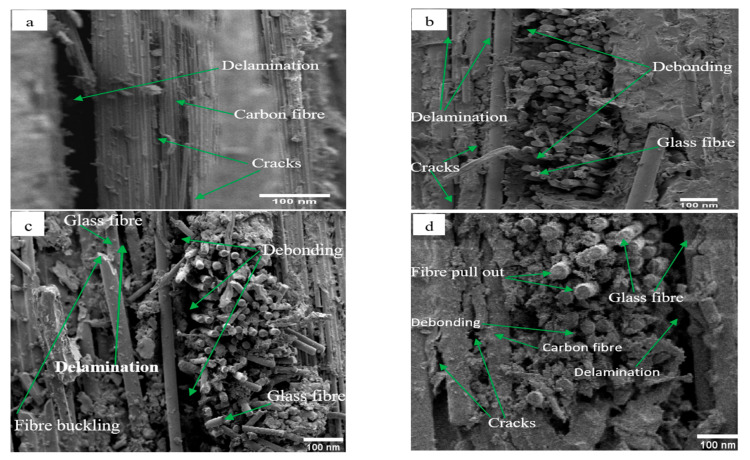
SEM images of bidirectional carbon (**a**), glass (**b**), glass–carbon (**c**), and glass–carbon–glass (**d**) composite laminates during room temperature tests.

Figure 8 shows the failure properties of C, G, GC, and GCG laminates obtained after the three-point bending tests. In the case of the pure carbon laminate, carbon fibre was arranged on the compressive and tensile sides of the specimen at the fibre configurations of [0/90]_s_. It is clear from Figure 8a that the delamination failure mode occurred near the top layers. Additionally, large and small cracks were observed near the [90]_s_ fibre configurations. Similarly, the failure properties of the pure glass laminate at a fibre configuration of [0/90]_s_ are shown in Figure 8b. It can be seen that scattered cracks and delamination failure modes were observed near the top layer of the specimen, which was fibre configured at [0]_s_. On the other hand, debonding failures were observed in the fibre configurations of [90]_s_. Based on the SEM results, the delamination failure mode is the critical failure mode in the three-point bending tests, when the fibres in pure carbon and glass laminates are arranged in [0]_s_. A debonding failure type was observed between the [0]_s_ and [90]_s_ fibre configurations.

The failure modes of the hybrid composite laminates were assessed using SEM, and are shown in Figure 8c,d. It can be seen from Figure 8c that fibre buckling and delamination failure modes were observed on the glass–carbon laminates near the top layers, with the arrangement of the fibres at [0]_s_. Voids and debonding between the fibre and the matrix were observed between [0]_s_ and [90]_s_ fibre configurations. Figure 8d shows the failure properties of the glass–carbon–glass laminates obtained from the SEM results. It can be seen that scattered cracks and delamination failure modes were observed in the [0]_s_ fibre configuration. In particular, the failure modes were changed to fibre pull-out and debonding when the fibre configurations were changed to [90]_s_. Based on the observation of the SEM results, scattered cracks and delamination failure modes occurred on the fibre configuration at [0]_s_. Fibre pull-out and debonding failure modes occurred in the case of [90]_s_ fibre configurations. This may have been due to the difference in the flow rate of the epoxy resin in [0]_s_ and [90]_s_ fibres.

## 4. Statistical Analyses

### Analysis of Variance (ANOVA)

One-way ANOVA analysis was performed to determine the effect of temperature on the flexural properties of C, G, GC, and GCG laminates [39]. The ANOVA results obtained from flexural strength tests in all groups of laminates under different testing temperatures are presented in Table 8, Table 9, Table 10 and Table 11. In this study, four groups and five observations were considered for each laminate to perform the analysis. As shown in Table 8, Table 9, Table 10 and Table 11, the total sum of the squares (SS) was obtained by adding the sum of squares between groups and the sum of squares within the groups of each laminate under consideration. The number of degrees of freedom (df) between groups was found by subtracting one from the number of groups of each laminate, whereas the number of degrees of freedom within groups was found by subtracting one from the number of observations and then multiplying by each group of laminates. The mean squares (MS) on each targeted laminate were obtained by dividing the sum of squares between groups and the sum of squares within groups by the respective degree of freedom. In the case of F statistics, the values were obtained by dividing the mean squares (MS) between groups by the mean squares within a group. Meanwhile, *p* is a probability value and F crit is an indicator that corresponds to the *p*-values which, when F crit < F, indicates that the variables had a significant effect on the outcomes. As shown in Table 8, Table 9, Table 10 and Table 11, values of *p* < 0.05 (F crit < F) were obtained for the flexural strength tests of laminates under temperatures of 25–100 °C. This indicates that the contribution of temperature is statistically significant, and must be considered when evaluating the flexural strength properties of C, G, GC, and GCG laminates under increasing temperature conditions.

## 5. Comparison with Empirical Models

In this section, the storage modulus results obtained from DMA tests on C, G, GC, and GC laminates are compared with empirical models [40]. According to Gibson et al. [41], the temperature-dependent behaviour of FRP composite materials can be determined based on the following equation:

(1) $$E\left(T\right)=\frac{E_{u}+E_{r}}{2}-\frac{E_{u}-E_{r}}{2}tanh(k\left(T-T\prime \right))$$

where *E*(*T*) is the elastic modulus at a specified temperature *T*, *E_u_* is the elastic modulus at room temperature, and *E_r_* is the material’s relaxed modulus before decomposition. The value of *k* is determined by fitting data using regression analysis, while *T*′ is assumed as a value of temperature at the glass transition temperature of the polymer.

Additionally, an empirical model for the temperature-dependent behaviour of FRP composite materials was developed by Gu and Asaro [42]. Their empirical model is given by:

(2) $$E\left(T\right)=E_{u}{\left(1-\frac{T-T_{r}}{T_{ref}-T_{r}}\right)}^{g}$$

where *E*(*T*) is the elastic modulus at a specified temperature *T*, *E_u_* is the elastic modulus at room temperature, *T_ref_* is a temperature where the elastic modulus tends towards lower values, and *T_r_* and *g* are the value of temperature at room temperature and a power-law index value from 0 to 1, respectively.

In this study, the empirical models developed by Gibson et al. and Gu and Asaro were implemented to validate the storage modulus results obtained from the tests. The parametric values used by the two empirical models, such as k and g, were determined using an Excel solver by calibrating the test data and the predicted model to achieve a minimum square error. A regression analysis was carried out to achieve a minimum error value between the experimental results and empirical models, as illustrated in Equations (1) and (2).

**Figure 9:**
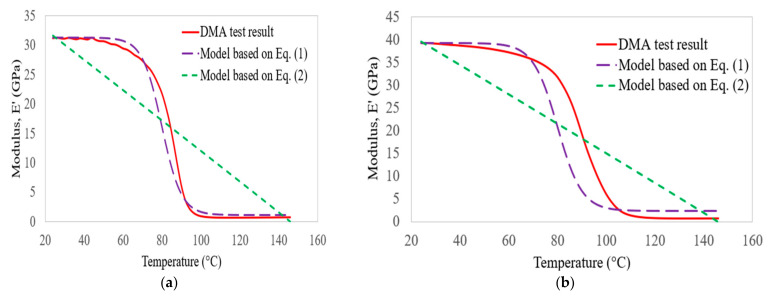
Comparison of carbon laminate storage modulus results with empirical models at 1 Hz (**a**) and 100 Hz (**b**).

Figure 9 shows the comparison between the empirical models developed by the authors of [41,42] and the storage modulus results of the bidirectional carbon laminates obtained from DMA tests. Based on the empirical equations given in Equations (1) and (2), the minimum square errors obtained at 1 Hz were about 0.85% and 7.39%, respectively. The errors were changed to about 3.96% and 6.16%, respectively, when the frequencies changed from 1 to 100 Hz. Based on the regression analysis, the empirical model given in Equation (1) was accurate for prediction.

**Figure 10:**
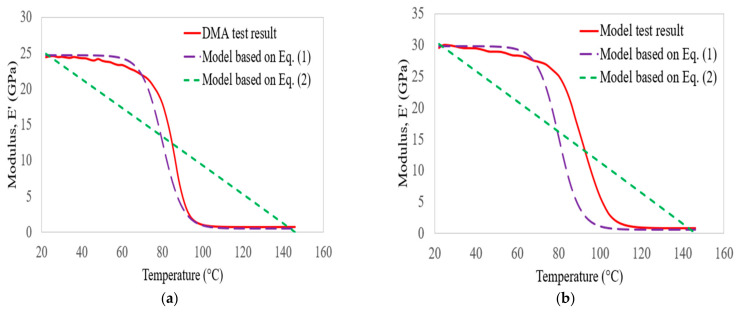
Comparison of glass laminate storage modulus results with empirical models at 1 Hz (**a**) and 100 Hz (**b**).

Figure 10 plots the storage modulus of bidirectional glass laminates with the predicted empirical models developed by Gibson et al. [41] and Gu and Asaro [42] subjected to increasing temperature and frequency. At a frequency of 1 Hz, the minimum square errors obtained using Equations (1) and (2) were about 1.13% and 7.52, respectively. Meanwhile, the errors were about 1.42% and 6.34%, respectively, as the frequencies changed from 1 to 100 Hz. In this case, the empirical model developed by Gibson et al. [41] was accurate for predicting the storage modulus results in the specified temperature and frequency range.

**Figure 11:**
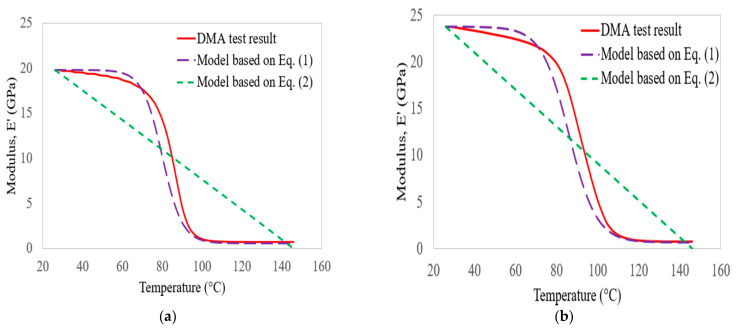
Comparison of glass–carbon laminate storage modulus results with empirical models at 1 Hz (**a**) and 100 Hz (**b**).

Figure 11 also plots the storage modulus of bidirectional glass–carbon composite laminates with the empirical models developed by Gibson et al. [41] and Gu and Asaro [42] as a function of increasing temperature and frequency. In this case, the minimum square errors between the test results and the empirical models were about 1.00% and 7.63, respectively, at a frequency of 1 Hz. At 100 Hz, the errors were about 4.81% and 6.43%, respectively. However, the empirical model developed by Gibson et al. [41] was better than that of Gu and Asaro [42] for prediction.

**Figure 12:**
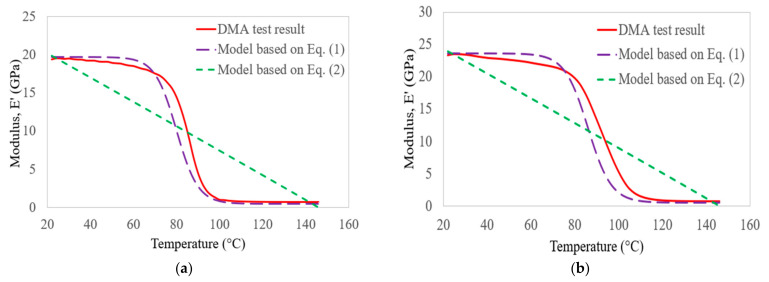
Comparison of glass–carbon–glass laminate storage modulus results with empirical models at 1 Hz (**a**) and 100 Hz (**b**).

The comparison between the storage modulus of bidirectional glass–carbon–glass laminates and the two empirical models given in Equations (1) and (2) is shown in Figure 12. The minimum square errors obtained based on the empirical models presented by Gibson et al. [41] and Gu and Asaro [42] were about 1.07% and 7.36%, respectively, at a frequency of 1 Hz. In the case of 100 Hz, the errors were about 1.16% and 5.98%, respectively. In all groups of laminates, the experimental results and the empirical models had good agreement. The errors were less than 10%, and the predictions were valid under the targeted temperature and frequency range. Based on least squares regression analysis, the empirical model developed by Gibson et al. [41] was the best-fitting empirical model for predicting the storage modulus of bidirectional C, G, GC, and GCG laminates at the targeted temperature and frequency range. However, further studies are necessary to revise the empirical parameters so as to obtain more accurate relationships with the storage modulus behaviour of FRP materials that have different glass transition temperatures.

## 6. Conclusions

This study is part of ongoing research focused on the mechanical performance of fibre-reinforced polymer composite materials that are available for modelling wind turbine blade structures applicable under different environmental conditions. Recently, the size of wind turbine blades has been increasing, and the candidate material for larger blade components such as skin and spar cap sections uses carbon fibres to reduce bending and torsion loads. This paper assessed the flexural performance, failure modes, and viscoelastic properties of bidirectional carbon, glass, glass–carbon, and glass–carbon–glass laminates for use on the skin section of the blades under increasing temperatures. Based on the experimental results, the following observations and conclusions were drawn:

1. Compressive and tensile stresses took place on the top and bottom parts of the laminates during the three-point bending tests. When glass fibres were in the top layers of the hybrid laminates, the strain at failure was optimised, which was better than that of pure carbon laminates. In particular, the flexural strength and stiffness properties of the hybrid laminates increased as the percentage of carbon fibre increased in the specimens, while the strain at failure of the hybrid laminates was reduced.
2. The storage modulus and flexural performance of all groups of composite laminates decreased as the testing temperature increased. In particular, the flexural modulus of the laminates decreased by 2–6% as the testing temperature increased from room temperature to 50 °C. This indicates that the flexural modulus of all groups of the targeted laminates slightly affected their mechanical properties below the glass transition temperature of the epoxy resin.
3. At a testing temperature of 100 °C, the flexural modulus of the laminates decreased from 62% to 73%, which occurred due to the weak properties of the epoxy matrix to transfer loads to the fibres.
4. The damping properties of the targeted laminates were assessed at increasing temperatures and frequencies. Pure carbon and glass laminates had the highest and lowest damping factors, respectively, while hybridisation of the two fibres—as well as increasing the amount of carbon fibre in the laminates—optimised the damping properties and increased their values.
5. The glass transition temperature of the epoxy matrix increased as the testing frequency changed from 1 to 100 Hz. This was obtained from the curves of the storage modulus, loss modulus, and damping factor, and occurred as a result of the additional time required to mobilise the epoxy molecules.
6. Delamination and debonding failure modes were the dominant failure modes in all groups of laminates. In particular, the delamination failure mode types occurred when the fibre orientation in the laminates was [0]_s_, whereas debonding of the fibre and the matrix was observed when the fibre orientation in the laminates was [90]_s_ or between [0]_s_ and [90]_s_. This might have been due to the difference in the flow rate of the epoxy resin in [0]_s_ and [90]_s_ fibres.

The experimental results obtained in this study provide a better understanding of the flexural performance, as well as the viscoelastic and failure properties, of bidirectional composite laminates under increasing temperature and frequency. These experimental results can be used to understand the failure types in the skin section of the composite wind turbine blade structures. Finally, the storage modulus results were compared with two empirical models that were important to reduce the material and testing costs. The empirical model developed by Gibson et al. was an accurate model to predict the storage modulus of all groups of laminates under the targeted temperature and frequency range. However, there was a mismatch between the calibrated test data and the values of the coefficients to fit properly with the other empirical model. More research is needed to revise the empirical parameters used to predict the storage modulus behaviour of FRP composite material in glassy, rubbery, and decomposed states.

## Figures and Tables

**Table 1:** Properties of carbon fibre, glass fibre, and epoxy resin at room temperature [36].

Materials	Elastic Modulus (GPa)	Tensile Strength (MPa)	Density (kg/m^3^)
T-300 carbon	230	3530	1760
E-glass	72.5	2350	2570
Epoxy	3.3	69.9	1020

**Table 2:** Designation of hybrid and non-hybrid laminates tested at different temperatures.

Laminate	Number of Layers	Temperature (°C)				Average Thickness (mm)	Hybrid Ratio
		25	50	75	100		
Carbon	C14	25/C	50/C	75/C	100/C	4.55 (C = 4.55, G = 0)	0
Glass	G12	25/G	50/G	75/G	100/G	4.55 (G = 4.55, G = 0)	1
Glass–carbon	G7C7	25/GC	50/GC	75/GC	100/GC	4.54 (G = 2.34, C = 2.20)	0.52
Glass–carbon–glass	G4C4G6	25/GCG	50/GCG	75/GCG	100/GCG	4.60 (G = 3.32, C = 1.28)	0.72

**Table 3:** Flexural strength (F_s_) and modulus (E) properties of bidirectional carbon laminates under different temperatures.

Laminates	Temperatures (°C)	F_s_ (MPa)	F_s_/ F_s,25_	SD	CV (%)	E (GPa)	E/ E_25_	SD	CV (%)
Carbon	25	763.63	1	23.61	3.09	84.64	1	0.72	0.85
	50	569.51	0.75	41.97	7.37	82.36	0.97	1.80	2.19
	75	262.14	0.34	15.11	5.76	51.38	0.61	3.78	7.35
	100	68.14	0.09	3.59	5.27	25.49	0.30	1.00	3.92

**Table 4:** Flexural strength (F_s_) and modulus (E) properties of bidirectional glass laminates under different temperatures.

Laminates	Temperatures (°C)	F_s_ (MPa)	F_s_/ F_s,25_	SD	CV (%)	E (GPa)	E/ E_25_	SD	CV (%)
Glass	25	502.76	1	18.25	3.63	30.59	1	2.33	7.62
	50	364.27	0.72	28.68	7.87	30.02	0.98	1.13	3.77
	75	155.27	0.31	4.00	2.58	19.49	0.64	0.89	4.57
	100	53.96	0.11	3.34	6.19	11.17	0.37	0.62	5.55

**Table 5:** Flexural strength (Fs) and modulus (E)properties of bidirectional glass–carbon laminates under different temperatures.

Laminates	Temperatures (°C)	F_s_ (MPa)	F_s_/ F_s,25_	SD	CV (%)	E (GPa)	E/ E_25_	SD	CV (%)
Glass-carbon	25	754.45	1	13.68	1.81	53.22	1	2.08	3.91
	50	481.03	0.64	18.99	3.95	48.88	0.92	3.81	7.80
	75	156.79	0.21	6.60	4.21	27.53	0.52	1.39	5.05
	100	55.83	0.07	2.98	5.33	14.34	0.27	0.59	4.11

**Table 6:** Flexural strength (Fs) and modulus (E) properties of bidirectional glass–carbon–glass laminates under different temperatures.

Laminates	Temperatures (°C)	F_s_ (MPa)	F_s_/ F_s,25_	SD	CV (%)	E (GPa)	E/ E_25_	SD	CV (%)
Glass-carbon -glass	25	560.07	1	18.55	3.31	31.95	1	1.48	4.63
	50	457.06	0.82	24.08	5.27	31.78	0.99	0.64	2.01
	75	148.82	0.27	12.10	8.13	17.75	0.56	0.76	4.28
	100	52.51	0.09	1.17	2.23	11.98	0.37	0.37	3.09

**Table 7:** Mechanical responses of C, G, GC, and GCG laminates on storage modulus (E′), loss modulus (E″), tan delta, and Tg as a function of temperature and frequency.

Laminates	E′ Max (GPa)	E′ at T_g_ (GPa)	E″ at Start (GPa)	E″ at T_g_ (GPa)	Peak Height	T_g_ on Tan Delta (°C)	T_g_ on E′ (°C)	T_g_ on E″ (°C)
C (1 Hz)	31.32	19.64	0.88	3.63	0.74	94	82	88
C (10 Hz)	31.31	20.78	0.73	3.66	0.75	100	84	90
C (100 Hz)	39.30	25.74	4.56	5.22	0.77	104	86	92
G (1 Hz)	19.57	11.14	0.51	2.42	0.59	94	82	88
G (10 Hz)	19.65	13.77	0.46	2.60	0.60	100	84	90
G (100 Hz)	23.55	17.96	2.19	3.48	0.63	106	84	94
GC (1 Hz)	24.61	17.98	0.46	3.02	0.68	94	80	88
GC (10 Hz)	24.68	18.65	0.42	3.24	0.68	100	82	90
GC (100 Hz)	29.60	22.52	2.82	4.34	0.70	106	84	94
GCG (1 Hz)	19.77	14.27	0.55	2.44	0.62	94	80	86
GCG (10 Hz)	19.85	15.04	0.47	2.59	0.63	100	82	92
GCG (100 Hz)	23.77	18.04	2.22	3.43	0.65	106	84	94

**Table 8:** Resulting ANOVA table for carbon laminates under testing temperatures of 25–100 °C.

Source of Variation	SS	df	MS	F	*p*-Value	F Crit
Between groups	1,445,473.90	3	481,824.63	752.78	$2.0\times 10^{-17}$	3.24
Within groups	10,240.96	16	640.06			
Total	1,455,714.86	19				

**Table 9:** Resulting ANOVA table for glass laminates under testing temperatures of 25–100 °C.

Source of Variation	SS	df	MS	F	*p*-Value	F Crit
Between groups	614,499.49	3	204,833.16	692.92	$3.9\times 10^{-17}$	3.24
Within groups	4729.75	16	295.61			
Total	619,229.24	19				

**Table 10:** Resulting ANOVA table for hybrid glass–carbon laminates under testing temperatures of 25–100 °C.

Source of Variation	SS	df	MS	F	*p*-Value	F Crit
Between groups	1,529,599.56	3	509,866.52	3877.3	$4.2\times 10^{-23}$	3.24
Within groups	2103.99	16	131.50			
Total	1,531,703.55	19				

**Table 11:** Resulting ANOVA table for hybrid glass–carbon–glass laminates under testing temperatures of 25–100 °C.

Source of Variation	SS	df	MS	F	*p*-Value	F Crit
Between groups	879,814.95	3	293,271.65	900.26	$4.8\times 10^{-18}$	3.24
Within groups	5212.23	16	325.76			
Total	885,027.18	19				

## Data Availability

The data presented in this study are available in the article.
